# Supplementary material for: Integrative Care for Challenging Behaviors in People with Intellectual Disabilities to Reduce Challenging Behaviors and Inappropriate Psychotropic Drug Prescribing Compared with Care as Usual: A Cluster-Randomized Trial
Source: Int J Environ Res Public Health. 2024 Jul 20;21(7):950. doi: 10.3390/ijerph21070950 (PMC11277020; doi:10.3390/ijerph21070950)
Supplement: Supplementary file 1 [file ijerph-21-00950-s001.zip › ijerph-3009304-supplementary.pdf]

SPIRIT Figure S1. Content for the schedule of enrolment, interventions, and assessments

	STUDY PERIOD								
	**Enrollment**	**Allocation**	**Post-allocation (weeks)**						
**TIMEPOINT**	***$-t_2$***	***$-t_1$***	***0***	***8***	***16***	***24***	***32***	***40***	***52***
**ENROLLMENT:**									
**Eligibility screen**	X								
**Informed consent**	X								
**Allocation**		X							
**INTERVENTIONS:**									
Integrative care intervention			◆	—	—	—	—	◆	
Care as usual (control)			◆	—	—	—	—	◆	
**ASSESSMENTS:**									
**Demographics**			X						
**Etiology ID**			X						
**PAS-ADD**			X						
**ABC**			X	X	X	X	X	X	X
**BPI-PIMD**			X	X	X	X	X	X	X
**DDD; other information on PD use**			X	X	X	X	X	X	X
**CLE**			X						X
**Diagnosis ICPC**			X					X	
**Restrictive measures**			X			X			X
**Treatment interventions**			X					X	X
**TAPP**			X					X	X
**MEDS**			X		X	X		X	X
**SCOPA-AUT**			X		X	X		X	X
**Adverse life events**			X		X	X		X	X

ID=Intellectual Disability; PAS-ADD=Psychiatric Assessment Schedule Adults with Developmental Disabilities; ABC=Aberrant Behavior Checklist; BPI-PIMD= Behavior Problems Inventory Dutch version for people with profound or multiple disabilities; DDD= Defined Daily Dose; PD=Psychotropic Drugs; CLE=Checklist Life Events; ICPC=International Codes of Primary Care; TAPP= Tool Appropriate Psychotropic drug Prescribing; MEDS= Matson Evaluation Drugs side-effects Scale; SCOPA-AUT=Scales for Outcomes in Parkinson disease -Autonomic dysfunction.
